# IL-34 is associated with the presence and severity of renal dysfunction and coronary artery disease in patients with heart failure

**DOI:** 10.1038/srep39324

**Published:** 2016-12-16

**Authors:** Qin Fan, Xiaoxiang Yan, Hang Zhang, Lin Lu, Qi Zhang, Fang Wang, Rui Xi, Jian Hu, Qiujing Chen, Wenquan Niu, Weifeng Shen, Ruiyan Zhang, Rong Tao

**Affiliations:** 1 Department of Cardiology, Rui Jin Hospital, Shanghai Jiaotong University School of Medicine, Shanghai, P.R. China; 2 Institute of Cardiovascular Diseases, Shanghai Jiaotong University School of Medicine, Shanghai, P.R. China; 3 State Key Laboratory of Medical Genomics, Ruijin Hospital, Shanghai Jiaotong University School of Medicine, Shanghai, P.R. China

## Abstract

Pro-inflammatory mediators are identified in patients with heart failure (HF), some of which may be used as biomarkers with diagnostic or prognostic value. As an additional ligand of Colony Stimulating Factor-1 Receptor (CSF-1R), interleukin-34 (IL-34) has been identified as a pro-inflammatory cytokine participating in chronic heart failure (CHF). However, the potential impact of IL-34 in CHF complications remains unknown. In order to determine the clinical significance of serum IL-34 in CHF patients, especially those with kidney dysfunction and coronary artery disease (CAD) comorbid conditions, serum IL-34 was measured in 510 consecutive patients with CHF in a cross-sectional study. The present study demonstrated that higher serum IL-34 levels were associated with poorer renal function and more severe anemia in patients with CHF. After adjusting for age, gender, conventional risk factors, and other significant covariates, IL-34 positively correlated with the presence and severity of renal dysfunction (as measured by eGFR and cystatin C) on multivariable linear and logistic regression analysis. IL-34 was also demonstrated to be an independent risk factor for CAD among HF patients. In conclusion, elevated serum IL-34 levels were demonstrated to be independently associated with renal insufficiency and CAD in patients with CHF, regardless of the systolic function.

Despite the progress achieved in its management so far, the prognosis of patients with heart failure (HF) remains poorer than that of most cancers^1^. Complication of heart failure such as renal dysfunction and coronary artery lesions could further aggravate the process of HF^2^. Pro-inflammatory cytokines are implicated in the initiation and progression of heart failure and its complications, and are known to provide important information regarding the pathogenesis, risk stratification, diagnosis, and therapeutic monitoring of heart failure, including in patients with either reduced or preserved ejection fraction^3^,^4^.

Interleukin-34 (IL-34), a cytokine identified in 2008, is expressed in several organs including the heart, brain, lung, liver, kidney, spleen and colon^5^. It was first described as an additional ligand of Colony Stimulating Factor-1 Receptor (CSF-1R). IL-34 has functions similar to CSF-1^5^, principally pertaining to the regulation of differentiation, proliferation, and survival of mononuclear phagocytes such as monocytes, macrophages and osteoclasts^6^, as well as stimulating the release of other pro-inflammatory cytokines^7^. IL-34 was found to be associated with inflammation process seen in diseases such as rheumatoid arthritis (RA)^8^, inflammatory bowel disease (IBD)^9^, and Sjogren’s syndrome^10^. Recent evidence demonstrated a marked renal tubular expression of IL-34 during ischemic injury in animal models, suggesting its role in mediating renal dysfunction, modulating inflammation^11^ and transplant tolerance^12^. Serum IL-34 was also found to be associated with heart failure in our previous study. However, little is known about the role and clinical significance of IL-34 in renal function and coronary artery lesions during heart failure. Therefore, the present study aimed to analyze the association of IL-34 with comorbid conditions of HF, including chronic kidney disease (CKD) and coronary artery disease (CAD).

## Results

### Baseline characteristics

A total of 510 patients with heart failure were enrolled for the present study. All patients were separated into three tertiles of patients with low (<61.40 pg/ml), median (61.40 pg/ml–97.39 pg/ml) and high (≥97.39 pg/ml) IL-34 levels (Table 1). Compared to subjects with lower IL-34, patients with higher IL-34 tended to be older (p = 0.016), however, with no significant difference between male and female patients. The levels of N-terminal pro brain natriuretic peptide (NT-proBNP), cystatin C and creatinine were significantly elevated in the higher IL-34 group compared to the lower group, and estimated glomerular filtration rate (eGFR) and hemoglobin was significantly reduced in the upper tertile of serum IL-34. However, the parameters of echocardiography including left ventricular ejection fraction (LVEF), left ventricular end-diastolic diameter (LVEDd) and left ventricular end-systolic diameter (LVESd) did not show remarkable difference.

### IL-34 as an independent risk factor for renal dysfunction in CHF patients

**Figure 1:**
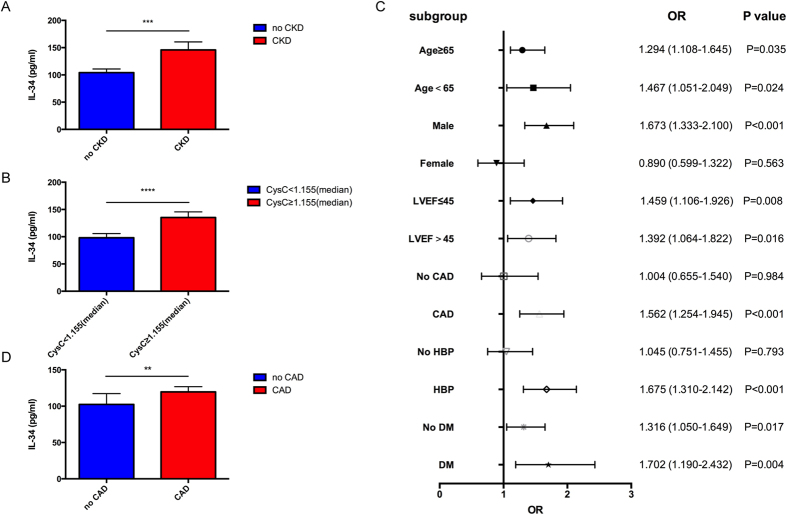
Association of serum IL-34 with renal dysfunction and CAD in patients with heart failure. (**A**) Comparison of serum IL-34 levels between heart failure patients with or without kidney dysfunction. Among all patients with heart failure, serum IL-34 levels are significantly higher in those with renal dysfunction than those without. (**B**) Serum IL-34 levels are significantly higher in HF patients with higher cystatin C level than those with lower cystatin C level, when median cystatin C is considered as a cut-off value. (**C**) Forrest plots (unadjusted) to analyze the predictive value of IL-34 for CKD in different subgroups of patients with heart failure. IL-34 is entered as a log transformed continuous variable. (**D**) Serum IL-34 levels are significantly higher in HF patients with CAD compared to those without CAD. **p = 0.0012, ***p < 0.001, ****p < 0.0001.

In patients with HF, serum IL-34 levels were not significantly different between patients with and without hypertension (117.64 ± 7.86 pg/mL vs. 113.59 ± 11.03 pg/mL, p = 0.764), as well as those with and without diabetes mellitus (106.15 ± 8.90 pg/mL vs. 122.04 ± 8.67 pg/mL, p = 0.202), indicating that there was no relationship between IL-34 and the presence of hypertension or diabetes mellitus. However, among HF patients, serum IL-34 levels were significantly higher in those combined with kidney dysfunction than those without (145.87 ± 14.68 pg/mL vs. 104.24 ± 6.65 pg/mL, p = 0.001) (Fig. 1A). IL-34 levels were also significantly higher in patients with cystatin C ≥ 1.550 (median) compared to those with cystatin C < 1.550 (135.26 ± 10.38 pg/mL vs. 98.00 ± 7.75 pg/mL, p < 0.0001) among patients with HF (Fig. 1B). When all HF patients were divided into tertiles according to IL-34 levels, the upper tertile had a significantly higher proportion of CKD patients compared to the lowest tertile (39.4% vs. 21.2%, p = 0.001).

Simple bivariate correlation analysis revealed that IL-34 negatively correlated with eGFR (r = −0.205, p < 0.001) and hemoglobin (Hb) (r = −0.169, p < 0.001), and positively correlated with cystatin C (r = 0.249, p < 0.001) and creatinine (r = 0.171, p < 0.001). Moreover, IL-34 was significantly associated with eGFR (β = −0.200, p < 0.001) and cystatin C (β = 0.199, p < 0.001) in univariate linear regression models. Multivariable linear regression analysis further demonstrated an independent correlation between IL-34 and eGFR (β = −0.152, p = 0.001) and log cystatin C (β = 0.118, p = 0.006), respectively, after adjusting for age, gender, body mass index (BMI), hypertension, diabetes, hemoglobin, albumin, high sensitivity C reactive protein (hsCRP) and NT-proBNP (Table 2).

Univariate logistic regression analysis revealed that IL-34, NT-proBNP, hsCRP, hemoglobin, albumin, age and New York Heart Association (NYHA) class significantly correlated with the presence of CKD. Furthermore, the multivariable regression models demonstrated significant independent correlation between serum IL-34 and the presence of CKD (odds ratio [OR]: 1.277; 95% confidence interval [CI]: 1.024–1.593, p = 0.030) (Table 3).

To further establish the correlation between IL-34 and CKD, we separately analyzed IL-34 as a log-transformed standardized continuous variable, as an ordinal variable with values expressed as tertiles, and as a categorical variable by using the lowest tertile as a reference value. Each of the models confirmed that IL-34 was an independent predictor for the presence of CKD among patients with HF, either adjusted for age and gender, or adjusted for the full model including age, gender, BMI, hypertension, diabetes mellitus, hsCRP, NT-proBNP, hemoglobin, albumin and NYHA functional class (Table 3).

On the other hand, subgroup analysis by using a logistic regression model revealed IL-34 to be a better predictor for worsening renal function in male patients with older age. The predictive value was also better in those concomitant coronary artery disease, hypertension, or diabetes mellitus. Moreover, IL-34 could predict the risk of kidney dysfunction between both patients with reduced and preserved ejection fraction. (Fig. 1C).

### IL-34 is independently associated with the presence and severity of CAD in patients with HF

In the present study, HF patients with CAD had higher IL-34 levels compared to those without CAD (115.68 ± 6.28 pg/mL vs 92.34 ± 6.64 pg/mL, p = 0.001) (Fig. 1D). Serum IL-34 levels were significantly associated with the CAG Gensini score (r = 0.176, p < 0.001) on simple linear correlation analysis, indicating that patients with higher serum IL-34 levels tended to have more severe coronary artery disease. Moreover, univariate and multivariable logistic regression analyses demonstrated that serum IL-34 remained a significant independent risk factor for CAD among HF patients, as well as when adjusted for age, gender, BMI, smoking, history of hypertension, diabetes mellitus, dyslipidemia, statin use, and laboratory tests including triglyceride, total cholesterol, low-density lipoprotein (LDL), glycosylated hemoglobin (HbA1c), hsCRP (Table 4).

## Discussion

This observational study demonstrates that in patients with chronic heart failure, increased serum levels of IL-34 were independently associated with the presence of kidney dysfunction and coronary artery disease, as well as their severity.

Renal dysfunction is common in HF patients and plays a critical role in the progression, and prognosis of heart failure as well as coronary heart disease^13^,^14^. A landmark analysis that included 1906 patients with HF demonstrated eGFR as the most powerful predictor of mortality exceeding the significance of left ventricular ejection fraction and NYHA functional class, establishing its role as a biomarker for renal dysfunction especially predicting poor outcome in patients with heart failure^15^. Another biomarker, cystatin C, also appears to be sensitive in the early detection of kidney dysfunction in HF patients^16^,^17^. In the present study, serum levels of IL-34 were found to be independently associated with renal biomarkers including eGFR and cystatin C among patients with HF, suggesting that IL-34 may play an important role in the process of heart failure mainly owing to its effect on renal function.

However, it is unclear whether IL-34 plays a maladaptive or a compensatory role in the pathogenesis of renal impairment, or only reflects the underlying renal dysfunction in patients with heart failure. On the basis of several experimental studies, it is reasonable to conclude that IL-34 has a participatory role in the pathophysiology of renal impairment, in addition to reflecting the severity of heart failure.

First, IL-34, generated by renal tubule epithelial cells (TECs), is up-regulated in kidney and serum during ischemia in a murine model of kidney ischemic reperfusion injury. Its overexpression is known to promote renal dysfunction in both the acute and chronic phases by the following two mechanisms: enhancing intra-renal macrophage proliferation and elevating bone marrow myeloid cell proliferation. These responses result in an increase in the circulating monocytes attracted to the kidney by chemokines^11^. These results indicate that IL-34 contributes to kidney dysfunction during ischemia. The reduced cardiac output in heart failure is principally responsible for low renal blood flow and increased renal venous pressure, and is one of the major determinants of renal impairment^18^. Renal destruction secondary to low renal blood flow is similar to that with ischemic injury, which in part explains the reason why serum IL-34 is up-regulated in heart failure-induced kidney dysfunction. However, this mechanism needs to be further explored.

Second, the role of IL-34 as an inflammatory mediator has been suggested by its ability to induce pro-inflammatory cytokines and chemokines such as IL-6, IL-8 and CCL2 in human whole blood^7^. Increased IL-34 levels are seen along with the increased expression of tumor necrosis factor alpha (TNF-α), IL-1β and IL-17, which are involved in regulating monocyte- and/or macrophage-induced inflammation associated with tubular destruction and lupus nephritis^10^,^19^. Thus, IL-34 may contribute to kidney function deterioration by its pro-inflammatory effects.

On the other hand, our present study demonstrated that higher serum IL-34 levels were associated with more severe coronary artery disease, which is in accordance with the results of a previous study with limited sample size that including only those subjects who had CAD^20^. Furthermore, another study illustrated that serum IL-34 may be superior to CRP as a potential inflammatory biomarker predicting the risk of vascular diabetic complications^21^. However, all these studies excluded patients with cardiac dysfunction or renal dysfunction, while our present study included those patients, thus expanding the scope of application of IL-34 as a predictive biomarker for CAD.

As discussed above, IL-34 may contribute to ischemic myocardial injury and atherosclerosis mainly owing to its function as a regulator of pro-inflammatory cytokines and innate immunity^11^,^22^. Moreover, pro-inflammatory cytokines can in turn induce increased expression and secretion of IL-34^23^, suggesting the presence of crosstalk between IL-34 and other cytokines. These findings support the role of an inflammatory process contributing to CAD. Segaliny *et al*. demonstrated that IL-34 could result in an *in vitro* increase in monocyte/CD34^+^ cell adhesion to activated human umbilical vein endothelial cell (HUVEC)–monolayers under physiological shear-stress conditions^24^. Hence it is also possible that IL-34 plays a part in CAD, partly by regulating mononuclear phagocyte adhesion to the endothelium, angiogenesis and macrophage recruitment.

Our present study suggested that IL-34 affect the severity of heart failure mainly through its involvement in the process of renal dysfunction during heart failure, and may influence the prognosis of heart failure, although it has not been shown to directly impair the process of cardiac remodeling, fibrosis or myocardial contraction. Second, an experimental study demonstrated that macrophages stimulated by IL-34 expressed galectin-3^25^. It is well known that galectin-3 is an important marker of cardiac remodeling as well as cardiac diastolic dysfunction^26^, and higher concentrations of galectin-3 are associated with increased risk for incident HF and mortality in the community^27^,^28^. Thus, IL-34 may have a role to play in the pathogenesis of heart failure mediated by galectin-3. However, the direct impact of IL-34 on heart failure and the underlying mechanisms needs to be further studied.

The present study however has several limitations.

First, the study was performed in a single center as a cross-sectional study. A larger prospective cohort is required to accurately assess the predictive value of serum IL-34 in the development of worsening renal function (WRF), which is common in HF, as well as to analyze its prognostic value in patients with heart failure^29^. In addition, both *in vivo* and *in vitro* studies are required to understand the precise mechanisms underlying the observations made in our present study, and to verify their clinical significance. Second, in order to confirm the effect of IL-34 on tubular damage, it is necessary to analyze serum IL-34 levels together with some tubular damage markers such as Neutrophil Gelatinase Associated Lipocalin (NGAL) and Kidney Injury Molecule 1 (KIM-1) in future studies.

## Conclusions

In conclusion, the present study confirmed that circulating IL-34 levels were significantly increased in HF patients with kidney dysfunction, and in HF patients with CAD. IL-34 levels significantly correlated with eGFR, cystatin C, and CAG Gensini score. These findings also indicate that baseline serum IL-34 levels may serve as independent predictors for kidney dysfunction and CAD in patients with chronic heart failure regardless of the level of systolic function. The role of IL-34 in assessing the prognosis of heart failure needs to be assessed in future cohort studies.

## Methods

We first studied serum IL-34 in a cohort of chronic heart failure patients to investigate its association with other renal haemodynamic measurements such as eGFR and cystatin C.

### Participants

This was a cross-sectional study that consecutively enrolled a total of 510 patients with chronic heart failure. The group consisted of patients who satisfied the following criteria: stable chronic heart failure with ≥3 months of diuretics use, hospitalization for heart failure at least once within a year before enrollment, and NT-proBNP levels of over 300 pg/mL for patients in sinus rhythm, and 900 pg/mL for those with atrial fibrillation at baseline. Included patients had heart failure either with preserved ejection fraction (HFpEF) or with reduced ejection fraction (HFrEF), however, all HF patients should have cardiac structural changes at baseline proved by echocardiography, including either left atrial enlargement, left ventricle enlargement or left ventricle hypertrophy. Patients with acute coronary syndrome during the four weeks preceding assessment or with significant concomitant diseases such as cancer, infections or autoimmune diseases were excluded. Patients who had renal replacement therapy or heart transplantation were also excluded. All included subjects were of age 18 years or above.

Data recorded at enrollment included results of routine laboratory tests, and details regarding concomitant diseases and cardiac medications. For each patient, echocardiography was performed at the same day when taking blood samples or within a week. The study was approved by the ethics review committee of our institution and a prior signed informed consent was obtained from each patient. All experiments and methods were performed in accordance with relevant guidelines and regulations.

### Measurements

Fasting venous blood samples were obtained to measure the levels of serum IL-34, NT-proBNP, hsCRP (high sensitivity C reactive protein), creatinine, and cystatin C, along with other routine biochemical indices. Once collected, the samples were immediately immersed in ice and centrifuged within 30 minutes at 2000 rpm for 20 min to obtain platelet-poor serum. All samples were stored at −80 °C before analysis.

1. Renal function

Estimated glomerular filtration rate (eGFR) was calculated by using a modified Modification of Diet in Renal Disease (MDRD) equation as follows: eGFR_MDRD_ = 30849*serum creatinine (μmol/L)–1.154*age–0.203 [*0.742 if women]. Preserved and impaired renal function were defined as eGFR_MDRD_ ≥60 mL/min per 1.73 m^2^ and eGFR_MDRD_ <60 mL/min per 1.73 m^2^, respectively.

2. Coronary artery disease

The diagnosis of CAD included involvement of at least one vessel, which was defined as >50% narrowing of the luminal diameter on coronary angiography, with or without angina pectoris. Coronary angiography (CAG) score was calculated by using the CAG Gensini score^30^.

3. IL-34

Serum IL-34 was measured by using a ELISA (Human IL-34 Quantikine ELISA Kit, D3400, R&D Systems, USA) according to the manufacturer guidelines.

### Statistical analyses

Continuous variables were expressed as mean and standard error (SE) if normally distributed. Values that were not normally distributed were natural log transformed before analysis. Categorical data were summarized as proportions and frequencies. Continuous variables were compared using independent t-test and one-way ANOVA test. Categorical variables were compared using Chi-square test or Fisher’s exact test as appropriate. The relationships between variables were tested by simple linear regression analyses. Both univariate and multivariable linear as well as logistic regression models were conducted to determine the influence of baseline levels of IL-34 on decline in eGFR and elevation in cystatin C, and on the presence of CKD, respectively. The predictive value of IL-34 was adjusted for age, gender, BMI, history of hypertension, history of diabetes mellitus, hsCRP, NT-proBNP, hemoglobin, albumin and NYHA functional class. Logistic regression analysis was also performed to assess the independent predictive value of IL-34 for CAD among heart failure patients. Odds ratios (OR) were presented as unadjusted, adjusted for age and gender, and further adjusted for age, gender, BMI, smoking, history of hypertension, history of diabetes. dyslipidemia, total cholesterol, triglyceride, LDL, HbA1c, hsCRP and statin use, respectively. All statistical analyses were performed using SPSS. Statistical significance was set at 2-tailed, and a p value < 0.05 was considered statistically significant.

## Additional Information

**How to cite this article**: Fan, Q. *et al*. IL-34 is associated with the presence and severity of renal dysfunction and coronary artery disease in patients with heart failure. *Sci. Rep.* **6**, 39324; doi: 10.1038/srep39324 (2016).

**Publisher’s note:** Springer Nature remains neutral with regard to jurisdictional claims in published maps and institutional affiliations.

## Figures and Tables

**Table 1:** Baseline characteristics according to IL-34 tertiles in HF patients.

	IL-34 < 61.40 pg/mL (n = 170)	61.40 pg/ml ≤ IL-34 < 97.39 pg/mL (n = 170)	IL-34 ≥ 97.39 pg/mL (n = 170)	P value
Age (y)	62.75 ± 0.92	65.88 ± 0.90	66.09 ± 0.93	0.016
Male gender	135 (79.4)	139 (81.8)	131 (77.1)	0.562
Smokers	79 (46.5)	73 (43.0)	76 (44.7)	0.807
Alcohol users	38 (22.4)	42 (24.7)	36 (21.2)	0.732
BMI (kg/m^2^)	25.05 ± 0.26	24.30 ± 0.30	24.16 ± 0.26	0.048
Systolic blood pressure (mmHg)	126.84 ± 1.87	127.61 ± 1.62	128.24 ± 1.66	0.848
Diastolic blood pressure (mmHg)	74.74 ± 1.16	75.04 ± 1.08	75.32 ± 0.96	0.928
Heart rate (beats/min)	79.31 ± 1.14	79.46 ± 1.72	79.58 ± 1.02	0.985
Medical history				
• Diabetes mellitus	60 (35.3)	64 (37.6)	62 (36.5)	0.903
• Hypertension	106 (62.4)	116 (68.2)	112 (65.9)	0.517
• Dyslipidemia	56 (32.9)	58 (34.1)	43 (25.3)	0.160
• CAD	118 (69.4)	144 (84.7)	145 (85.3)	<0.001
• CKD	36 (21.2)	44 (25.9)	67 (39.4)	0.001
Echocardiography				
• LVEF (%)	43.65 ± 0.93	42.81 ± 0.82	45.72 ± 0.85	0.053
• LVEDd (mm)	59.66 ± 0.74	59.38 ± 0.61	59.13 ± 0.60	0.850
• LVESd (mm)	46.51 ± 0.84	46.23 ± 0.70	45.23 ± 0.70	0.447
• LAd (mm)	43.99 ± 0.53	43.68 ± 0.43	43.96 ± 0.41	0.871
Lab. Examination				
Hemoglobin (g/L)	134.02 ± 1.35	131.64 ± 1.30	126.79 ± 1.44	0.001
Fasting glucose (mmol/L)	6.17 ± 0.24	5.98 ± 0.16	5.75 ± 0.15	0.283
HbA1c (%)	6.53 ± 0.10	6.55 ± 0.11	6.44 ± 0.10	0.735
BUN (mmol/L)	6.86 ± 0.25	6.65 ± 0.24	7.38 ± 0.30	0.135
Creatinine (μmol/L)	90.57 ± 2.49	92.24 ± 2.29	116.13 ± 8.96	<0.001
Uric acid (μmol/L)	373.68 ± 10.46	379.25 ± 9.64	374.45 ± 10.09	0.914
eGFR_MDRD_ (ml/min/1.73^2^)	76.80 ± 1.66	74.71 ± 1.80	66.93 ± 1.78	<0.001
Cystatin C (mg/L)	1.18 ± 0.03	1.28 ± 0.03	1.52 ± 0.09	<0.001
Total cholesterol (mmol/L)	4.09 ± 0.08	3.89 ± 0.08	3.92 ± 0.08	0.157
Triglyceride (mmol/L)	1.61 ± 0.09	1.54 ± 0.06	1.38 ± 0.05	0.064
LDL-C (mmol/L)	2.50 ± 0.07	2.32 ± 0.06	2.35 ± 0.07	0.096
HDL-C (mmol/L)	1.01 ± 0.02	0.96 ± 0.02	1.00 ± 0.02	0.294
hsCRP (mg/L)	14.23 ± 2.85	20.80 ± 3.80	21.14 ± 3.63	0.222
NT-proBNP (pg/mL)	2638.08 ± 311.30	3111.79 ± 410.31	3774.87 ± 389.58	0.011
Medications				
• ACEI/ARB	122 (71.8)	137 (80.6)	127 (74.7)	0.155
• β-blocker	147 (86.5)	147 (86.5)	140 (82.4)	0.469
• Spironolactone	77 (45.3)	80 (47.1)	76 (44.7)	0.902
• Diuretics	71 (41.8)	83 (48.8)	72 (42.4)	0.348
• Antiplatelet drugs	133 (78.2)	148 (87.1)	139 (81.8)	0.100
• Nitrates	54 (31.8)	76 (44.7)	87 (51.2)	0.001
• Statins	120 (70.6)	144 (84.7)	132 (77.6)	0.008
• Hypoglycemic drugs	27 (15.9)	37 (21.8)	37 (21.8)	0.291

Data are presented as mean ± SE or n (%). BMI, body mass index; CAD, coronary artery disease; CKD, chronic kidney disease; LVEF, left ventricular ejection fraction; LVEDd, left ventricular end-diastolic diameter; LVESd, left ventricular end-systolic diameter; LAd, left atrium diameter; BUN, blood urea nitrogen; eGFR, estimated glomerular filtration rate; LDL, low-density lipoprotein; HDL, high-density lipoprotein; hsCRP, high sensitivity C reactive protein; NT-proBNP, pro-brain natriuretic peptide; ACEI, angiotensin-converting enzyme inhibitor; ARB, angiotensin receptor blocker.

**Table 2:** Univariate and Multivariable Linear Regression Models for eGFR and cystatin C in patients with heart failure.

eGFR	Univariate analysis		Multivariable analysis				Univariate analysis		Multivariable analysis		
	β	P value	β	Part.cor.	P value	Cystatin C	β	P value	β	Part.cor.	P value
Log IL-34	−0.200	<0.001	−0.152	−0.165	0.001	**Log IL-34**	0.199	<0.001	0.118	0.135	0.006
Age	−0.310	<0.001	−0.237	−0.243	<0.001	**Age**	0.286	<0.001	0.159	0.172	<0.001
Gender	0.071	0.108	—	—	—	**Gender**	0.078	0.080	0.180	0.205	<0.001
BMI	0.106	0.018	—	—	—	**BMI**	−0.156	0.001	—	—	—
hsCRP	−0.126	0.007	—	—	—	**hsCRP**	0.141	0.003	—	—	—
NT-proBNP	−0.332	<0.001	−0.239	−0.245	<0.001	**NT-proBNP**	0.358	<0.001	0.243	0.261	<0.001
Hb	0.213	<0.001	—	—	—	**Hb**	−0.323	<0.001	−0.260	−0.273	<0.001
Alb	0.193	<0.001	—	—	—	**Alb**	−0.221	<0.001	—	—	—
NYHA	−0.186	<0.001	—	—	—	**NYHA**	0.212	<0.001	—	—	—
HBP	−0.084	0.059	—	—	—	**HBP**	0.149	0.001	0.100	0.116	0.018
DM	0.076	0.088	—	—	—	**DM**	−0.038	0.403	—	—	—

IL-34 is analyzed as a log transformed continuous variable. Model: adjusted for age, gender, body mass index, history of hypertension, history of DM, hsCRP, NT-proBNP, hemoglobin, albumin, NYHA class and medication. BMI, body mass index; hsCRP, high sensitivity C reactive protein; NT-proBNP, pro-brain natriuretic peptide; Hb, hemoglobin; Alb, albumin; NYHA, New York Heart Association; HBP, high blood pressure; DM, diabetes mellitus.

**Table 3:** Univariate and Multivariable Logistic Regression Models for CKD in HF patients.

	Unadjusted OR	P value	Adjusted for Model 1 OR	P value	Adjusted for Model 2 OR	P value
Log IL-34 per SD	1.423 (1.177–1.720)	<0.001	1.349 (1.109–1.640)	0.003	1.277 (1.024–1.593)	0.030
IL-34 tertiles	1.574 (1.236–2.004)	<0.001	1.495 (1.163–1.923)	0.002	1.435 (1.095–1.881)	0.009
1st tertile	1 (ref)		1 (ref)		1 (ref)	
2nd tertile	1.300 (0.786–2.150)	0.307	1.127 (0.669–1.899)	0.653	1.317 (0.747–2.321)	0.341
3rd tertile	2.421 (1.499–3.911)	<0.001	2.162 (1.315–3.554)	0.002	2.036 (1.183–3.502)	0.010

IL-34 is analyzed as a log transformed continuous variable, an ordinal variable divided according to tertiles of IL-34, and a categorical variable using the lowest tertile as reference. Model 1: adjusted for age and gender. Model 2: adjusted for age, gender, body mass index, history of hypertension, history of DM, hsCRP, NT-proBNP, hemoglobin, albumin, NYHA class and medication. As a continuous variable, OR is shown as per 1 SD (standard deviation). CKD, chronic kidney disease; OR, odds ration; IL-34, interleukin 34; DM, diabetes mellitus; hsCRP, high sensitivity C reactive protein; NT-proBNP, pro-brain natriuretic peptide; NYHA, New York Heart Association.

**Table 4:** Univariate and Multivariable Logistic Regression Models for CAD in HF patients.

	Unadjusted OR	P value	Adjusted for Model 1 OR	P value	Adjusted for Model 2 OR	P value
Log IL-34	1.428 (1.115–1.829)	0.005	1.396 (1.083–1.798)	0.010	1.351 (1.005–1.816)	0.047
IL-34 tertiles	1.660 (1.259–2.188)	<0.001	1.654 (1.242–2.202)	0.001	1.627 (1.131–2.341)	0.009
1st tertile	1 (ref)		1 (ref)		1 (ref)	
2nd tertile	2.441 (1.437–4.146)	0.001	2.242 (1.292–3.889)	0.004	2.078 (1.001–4.313)	0.050
3rd tertile	2.556 (1.496–4.366)	0.001	2.583 (1.472–4.534)	0.001	2.579 (1.255–5.297)	0.010

IL-34 is analyzed as a log transformed continuous variable, an ordinal variable divided according to tertiles of IL-34, and a categorical variable using the lowest tertile as reference. Model 1: adjusted for age and gender. Model 2: adjusted for age, gender, smoke, body mass index, history of hypertension, history of DM, history of dyslipidemia, triglyceride, total cholesterol, LDL-c, HbA1c, hsCRP and statin use. As a continuous variable, OR is shown as per 1 SD (standard deviation). OR, odds ratio; HF, heart failure; DM, diabetes mellitus; LDL-c, low density lipoprotein-cholesterol; HbA1c, glycosylated hemoglobin; hsCRP, high sensitivity C reactive protein.
